# Angiotensin II type 1 receptor blocker-induced immune thrombocytopenia: a case report

**DOI:** 10.1186/1752-1947-7-183

**Published:** 2013-07-15

**Authors:** Dhiren K Patel, Nikhil Bilkha, David Schnee

**Affiliations:** 1 VA Boston Healthcare System, 940 Belmont Street, Brockton, MA 02301, USA; 2 Massachusetts College of Pharmacy and Health Sciences, 179 Longwood Avenue, Boston, MA 02115, USA; 3 VA Boston Healthcare System, 150 South Huntington Avenue, Boston, MA 02130, USA

**Keywords:** Drug-induced thrombocytopenia, Adverse drug reactions, Losartan, Valsartan, Angiotensin receptor blocker

## Abstract

**Introduction:**

The development of thrombocytopenia after a dose increase in losartan and subsequently after switching the patient to valsartan is reported.

**Case presentation:**

A 61-year-old Caucasian man presented with epistaxis and gingival bleeding of three weeks duration. Laboratory evaluation revealed a hemoglobin level of 144g/L, a leukocyte count of 16.2×10^9^ cells/L (72.51% neutrophils, 20.1% lymphocytes, 6.8% monocytes, 0.4% eosinophils, 0.2% bands), and a platelet count of 15.0×10^9^ cells/L. Flow cytometry of his peripheral blood showed normal CD4:CD8 ratio and no evidence of any lymphoproliferative disorder. A peripheral smear showed decreased platelets with a few areas of clumping. Four weeks before presentation to the emergency room, his losartan dose was increased to 100mg once daily due to continuously elevated blood pressure readings. He had been maintained on losartan 50mg once daily for five years and previous routine laboratory measurements revealed a baseline platelet count of 248.0×10^9^ cells/L.

The patient began receiving an oral prednisone taper and his platelet count returned to a stable value of >200×10^9^ cells/L. Because there was no other probable cause, he was thought to have developed immune thrombocytopenia from the increased losartan dose. Losartan was discontinued and one week later he was switched to valsartan 160mg once daily.

Forty-seven days after starting valsartan, the patient presented once again to the emergency room with intermittent epistaxis and gingival bleeding while brushing his teeth of two weeks duration. Laboratory measurement revealed a platelet count of 37×10^9^ cells/L. Valsartan was held and another prednisone taper was initiated. The patient’s platelet count recovered upon valsartan discontinuation and in four weeks, his platelet count improved to 214×10^9^ cells/L.

**Conclusions:**

A 61-year-old Caucasian man developed immune thrombocytopenia after an increase in losartan dose and developed immune thrombocytopenia again after he was switched to valsartan.

## Introduction

Drug-induced thrombocytopenia (DIT) is a phenomenon that is most frequently associated with heparin, but it has been attributed to many other agents. Clinicians often discontinue all medications when a patient presents with thrombocytopenia, making it difficult, if not impossible, to determine the causative agent when platelet counts improve [1]. This has resulted in an exhaustive list of drugs reported to cause thrombocytopenia despite an estimated incidence of DIT of <1% in patients receiving medications excluding heparin [1]. In this report, we describe the case of a patient who developed thrombocytopenia after an increase in losartan dose and subsequently upon initiation of valsartan.

We performed a MEDLINE literature search (1970 to present) using all possible combinations of the following key words: ‘angiotensin’, ‘thrombocytopenia’, ‘thrombocytopaenia’, ‘thrombopenia’, ‘azilsartan’, ‘candesartan’, ‘eprosartan’, ‘irbesartan’, ‘losartan’, ‘olmesartan’, ‘telmisartan’, and ‘valsartan’. Our search revealed only one case report in the literature published by Ada *et al*. in 2002 in the *Annals of Internal Medicine*[2]. This was a case of an 82-year-old woman who presented with epistaxis and easy bruising of two weeks duration. Two weeks before presentation she had stopped taking quinapril because of a cough and was switched to losartan. Her only other medications were nortriptyline and bumetanide, both of which she had taken for two years. Her platelet count returned to normal within one week after discontinuing losartan [2].

In addition to our MEDLINE search, we reviewed the prescribing information for all commercially available angiotensin II type 1 receptor blockers (ARBs) in order to capture cases of possible ARB-induced thrombocytopenia that have not been described in the literature. Currently there are eight ARBs available for use in the United States: azilartan (Edarbi™), candesartan (Atacand™), eprosartan (Teveten™), irebesartan (Avapro™), losartan (Cozaar™), olmesartan (Benicar™), telmisartan (Micardis™), and valsartan (Diovan™) [3-10]. One patient receiving candesartan and four patients receiving eprosartan were withdrawn from clinical trials after developing thrombocytopenia [4,5]. Thrombocytopenia has been reported as part of postmarketing surveillance for irbesartan and telmisartan, but the incidence has not been described [6,9]. Thrombocytopenia has been reported as part of postmarketing surveillance for valsartan and losartan but has been ‘reported rarely’ [7,10]. There have been no cases of thrombocytopenia described in the prescribing information for azilsartan and olmesartan [3,8]. Given this limited information, the incidence of probable ARB-induced thrombocytopenia is unclear.

## Case presentation

A 61-year-old, 116kg, 71 inch Caucasian man presented to the emergency room with epistaxis and gingival bleeding of three weeks duration. Upon physical examination, no other gross bleeding was apparent and he denied a history of similar bleeding events. He was hemodynamically stable, alert and oriented, and in no acute distress. Results of his heart and lung examinations were unremarkable. Laboratory evaluation revealed a hemoglobin level of 144g/L, a leukocyte count of 16.2×10^9^ cells/L (72.51% neutrophils, 20.1% lymphocytes, 6.8% monocytes, 0.4% eosinophils, 0.2% bands), and a platelet count of 15.0×10^9^ cells/L. Flow cytometry of his peripheral blood showed normal CD4:CD8 ratio and no evidence of any lymphoproliferative disorder. A peripheral smear showed decreased platelets with a few areas of clumping. Four weeks before presentation, his losartan dose was increased to 100mg once daily due to continuously elevated blood pressure readings. He had been maintained on losartan 50mg once daily for five years and previous routine laboratory testing revealed a baseline platelet count of 248.0×10^9^ cells/L.

**Figure 1:**
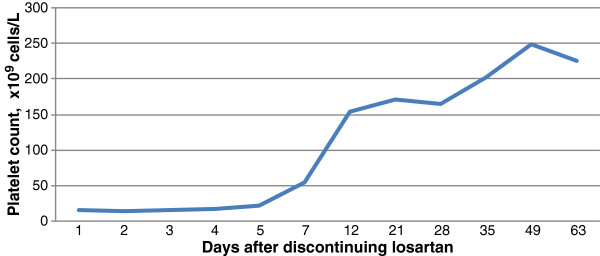
Platelet count following discontinuation of losartan.

The patient began receiving an oral prednisone taper and his platelet count returned to a stable value of >200×10^9^ cells/L (Figure 1). Because there was no other probable cause, he was thought to have developed immune thrombocytopenia from the increased losartan dose. Losartan was discontinued and one week later he was switched to valsartan 160mg once daily.

This patient’s past medical history was significant for primary hypertension, uncontrolled type 2 diabetes mellitus, gastroesophageal reflux disease, post-traumatic stress disorder, depression, bipolar disorder, and dyslipidemia. His past surgical history included splenectomy, right hand skin graft, ventral hernia repair, and a gunshot wound to the back of the neck. The patient had no prior history of hematologic abnormalities or vascular disease. His hepatic and renal functions were normal and stable. His medications included losartan 100mg once daily (increased from 50mg once daily four weeks before presentation), aspirin 325mg once daily, metformin 1000mg twice daily, atenolol 75mg once daily, simvastatin 20mg once daily, ranitidine 150mg twice daily, Novolin™ 70/30 insulin 75 units subcutaneously every morning and 85 units every evening, temazepam 15 to 30mg daily as needed at bedtime, and a multivitamin daily. He had been taking all of these medications for at least five years. He has no known drug allergies, but had a documented cough with lisinopril. The patient had no history suggestive of infection and no history of chemotherapy, radiation, or malignancy. He denied alcohol, tobacco, or illicit drug use and any sexually transmitted disease risk factors. He denied any family history of any bleeding diatheses.

**Figure 2:**
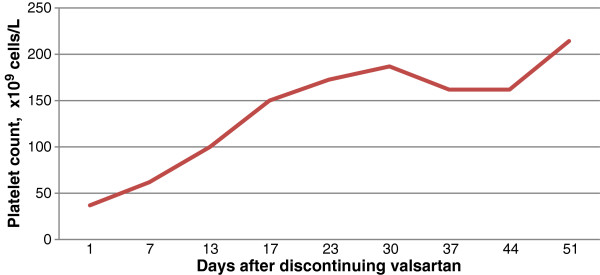
Platelet count following discontinuation of valsartan.

Forty-seven days after starting valsartan, the patient presented once again to the emergency room with intermittent epistaxis and gingival bleeding while brushing his teeth of two weeks duration. Laboratory measurement revealed a platelet count of 37×10^9^ cells/L. Valsartan was held and another prednisone taper was initiated. The patient’s platelet count recovered upon valsartan discontinuation and in four weeks, his platelet count improved to 214×10^9^ cells/L (Figure 2). To date, the patient’s platelet count has remained normal on follow-up evaluations.

## Discussion

A clear correlation exists between the two episodes of thrombocytopenia and ARB use in our patient. During his initial hospitalization, only his insulin doses were adjusted and no new agents were initiated. The patient had no other conditions associated with thrombocytopenia and had an unremarkable past medical history for bleeding or thrombocytopenia. At first, it was thought that thrombocytopenia was a result of losartan since his platelet count decreased after the dose was increased and his platelet count recovered when the drug was discontinued. However, there was further evidence for this reaction when the patient was switched to an alternative ARB, valsartan, and his platelet count decreased again. When valsartan was discontinued, his platelets returned to a normal level.

The Naranjo algorithm for adverse drug reaction assessment indicated a score of nine for this patient’s experience with ARBs [11]: thrombocytopenia occurred after either ARB was administered (+2); thrombocytopenia improved after either ARB was discontinued (+1); thrombocytopenia recurred when another ARB was administered (+2); no alternate causes on their own could have caused the reaction (+1); thrombocytopenia was more severe when the dose of losartan was increased (+1); thrombocytopenia occurred in a previous exposure to an ARB (+1); and thrombocytopenia was confirmed by objective evidence (that is, platelet count) (+1). As only one case report of ARB-induced thrombocytopenia has been reported in the literature and since the information on causality in the prescribing information is lacking, we opted for a conservative approach and did not add an additional point for the existence of conclusive reports of this reaction. Nevertheless, scores ≥9 using this algorithm are indicative of a definite association between the adverse reaction and the suspected drug [12].

In addition, we evaluated the casual relationship between ARB use and thrombocytopenia using a scale developed by George *et al*. that specifically evaluates the likelihood of DIT [13]. The four criteria of this scale are: 1) drug administration preceded thrombocytopenia; recovery from thrombocytopenia complete and sustained after the drug was discontinued, 2) other drugs administered prior to thrombocytopenia were continued or reintroduced after discontinuation of the suspected drug, 3) other etiologies of thrombocytopenia were excluded, 4) re-exposure to the drug resulted in recurrent thrombocytopenia. Because all four of these criteria were met in our patient, this would be a case of definite DIT.

There is a great deal of similarity between this case and the other existing case report of ARB-induced thrombocytopenia reported by Ada *et al*. 10 years ago [2]. Both patients presented with similar symptoms with laboratory results demonstrating thrombocytopenia that could only be attributed to ARB use. What differentiates our case is that the initial thrombocytopenic event was triggered by doubling the dose of losartan; there was no initial adverse reaction when our patient started losartan more than five years before. Because of the nephroprotective benefits of ARB therapy in diabetics as an alternative to an angiotensin-converting enzyme inhibitor [14,15], an alternative ARB, valsartan, was initiated. However, this resulted in the second case of thrombocytopenia that was discovered after the patient presented with similar bleeding symptoms as the initial event.

DIT is often an immune-mediated reaction and has many possible explanations [16]; however, accelerated platelet destruction caused by drug-dependent antibody formation is a viable explanation in the case of this patient. In the presence of a sensitizing drug, drug-dependent antibodies develop that are highly specific to the drug’s structure approximately one to two weeks after drug exposure [17]. These drug-dependent antibodies react with platelets via their fragment antigen-binding (Fab) domains, rather than their fragment crystallizable (Fc) domains [16]. This is in contrast to other immune-complex reactions, such as in heparin-induced thrombocytopenia where heparin-dependent antibodies react with platelets via their Fc domain, eventually resulting in thrombotic complications despite a low platelet count [18]. It is unknown why these drug-dependent antibodies target platelets instead of other cells such as leukocytes or erythrocytes [17].

Losartan was the prototype for the ARB class and was followed by valsartan; the only structural difference being replacement of the imidazole of losartan with an acylated amino acid [19]. Aside from this structural difference, losartan and valsartan have similar pharmacologic and pharmacokinetic profiles [19]. It is unclear whether antibody cross-reactivity is possible with losartan and valsartan; however, because their chemical structures are more similar compared with later ARBs such as telmisartan, it cannot be ruled out as a possibility.

Because of the very low reported incidence of ARB-induced thrombocytopenia, routine monitoring of platelets is probably not necessary. However, ARBs should be evaluated as potential causative agents in patients who present with idiosyncratic thrombocytopenia and are receiving ARB therapy. Based on our case, if a patient develops thrombocytopenia thought to be related to an ARB, the ARB should be discontinued, and an alternative antihypertensive agent, outside of the ARB class, should be initiated. Furthermore, patients should always be encouraged to report any signs of easy bruising or spontaneous bleeding when taking any drug, as these may be an early indication of DIT.

## Conclusions

A 61-year-old Caucasian man developed immune thrombocytopenia after an increase in losartan dose and developed immune thrombocytopenia again after he was switched to valsartan.

### Consent

Written informed consent was obtained from the patient for publication of this case report and any accompanying images. A copy of the written consent is available for review by the Editor-in-Chief of this journal.

## Competing interests

The authors declare that they have no competing interests.

## Authors’ contributions

All authors were equally responsible for data collection and writing of the manuscript. All authors read and approved the final manuscript.
